# Attribute encryption access control method of high dimensional medical data based on fuzzy algorithm

**DOI:** 10.1371/journal.pone.0317119

**Published:** 2025-03-27

**Authors:** Yonggang Huang, Teng Teng, Yuanyuan Li, Minghao Zhang

**Affiliations:** Information Management, The First Affiliated Hospital of Hebei North University, Zhangjiakou, China; Jaramogi Oginga Odinga University of Science and Technology, KENYA

## Abstract

The current approach to data access control predominantly utilizes blockchain technology. However, when dealing with high-dimensional medical data, the inherent transparency of blockchain conflicts with the necessity of protecting patient privacy. Consequently, this increases the risk of sensitive information exposure. To enhance patient privacy, a fuzzy encryption algorithm is employed. This prevents unauthorized access and decryption of sensitive medical data. Consequently, a high-dimensional medical data attribute encryption access control method based on fuzzy algorithm is proposed. Phase data and frequency data are utilized to assess the stability of medical data attributes. Additionally, the empirical mode decomposition method is applied to eliminate noise from these attributes. Using the key configuration of fuzzy encryption algorithm, high-dimensional medical data attributes with different security levels within the same field undergo encryption and decryption processes. Moreover, the trust degree of access behavior towards these data attributes is calculated to maintain security. After the medical users successfully log in, their access permissions are analyzed to effectively control the encrypted access permissions of high-dimensional medical users. The access request graph is established to effectively control encrypted access to high-dimensional medical data attributes. The experimental results showed that when the number of data attributes reached millions, the encryption access control time was still less than 60ms. The maximum encryption time was reduced by 21ms, and the anti-attack success rate was high during the application process. From the comparison of the maximum success rates, it can be seen that the success rate of this method in resisting attacks has increased by 8.5%.

## 1. Introduction

With the rapid development of medical information technology, an increasing number of medical institutions collect and store a vast amounts of high-dimensional medical data [1]. High-dimensional medical data refers to data collected in the medical field containing multiple characteristics (dimensions), including patient identity information (such as age, gender, race, family status), disease status (such as disease type, severity, course of disease), and drug use (such as drug type, dose, frequency) [2]. Medical data attributes can be broadly categorized into two types: qualitative and quantitative. Qualitative attributes are those that possess classification or labeling properties, such as gender and disease type, which describe categorical characteristics. Conversely, quantitative attributes are those that carry numerical values, like age and drug dosage, providing measurable quantities. Additionally, medical data attributes include “stage” and “frequency,” which specify the time frame and the rate of data collection, respectively. These attributes are essential for accurately recording the condition of patients at various stages [3]. However, the security and privacy protection of these high-dimensional medical data are confronting significant challenges. During the collection, processing, and integration of high-dimensional medical data, various noise interferences may arise. These include data errors stemming from human input mistakes and equipment malfunctions during the data collection phase. In the subsequent data cleaning and processing stage, algorithm or operational errors may emerge, thereby introducing additional noise. Moreover, inconsistencies in data sources during data fusion can also lead to noise interference. The primary impact of such noise is a decrement in data quality [4]. Noise interference makes high-dimensional medical data inaccurate and unreliable, affecting the accuracy of medical decision-making. In data analysis, if noise is not effectively eliminated, it can lead to incorrect conclusions and inaccurate predictions. In addition, noise increases the difficulty of data processing and analysis, reducing the interpretability and reliability of data [5]. The sharing and interoperability of medical data have enormous potential in promoting collaborative healthcare, facilitating medical research, and strengthening public health. However, this process is not without challenges, primarily concerning data security and privacy protection [6]. Therefore, it is urgent to study attribute encryption access control method for high-dimensional medical data. By combining encryption technology with access control mechanism, it ensures that only authorized users can access sensitive medical data, so as to achieve data security and privacy protection [7]. At the same time, given the unique characteristics of high-dimensional medical data, it is imperative to optimize and enhance aspects such as data dimension, spatial complexity, and computational overhead. Consequently, conducting a thorough analysis of encryption access control methods specifically tailored for the attributes of high-dimensional medical data holds significant practical importance [8]. It can not only protect the privacy and data security of patients, but also promote the sharing and utilization of medical data, which is beneficial for the development of medical research and clinical practice. At the same time, the research also has certain challenges. Medical data is often high-dimensional data, including various attributes and features. In this case, how to effectively encrypt and access control high-dimensional data to ensure data security and not affect the availability of data is a challenge. In the access control of medical data, it is necessary to carry out fine-grained control on the data according to user roles and authority [9]. In the realm of encryption, achieving precise access control presents a notable challenge. The objective is to guarantee that only legitimate users are granted the appropriate data access permissions. Simultaneously, it is imperative to safeguard sensitive data from unauthorized access [10]. Consequently, it is essential to enhance both data access efficiency and computing performance, while maintaining robust data security, to fulfill the practical requirements of the medical system.

## 2. Related work

With the rapid development of information technology, data has become an important asset for both enterprises and individuals. Encrypted access control can ensure the security of data during transmission and storage, preventing unauthorized access or leakage of data. Therefore, many domestic and foreign scholars have conducted research on data encryption access control methods. Boomija et al. [11] proposed a medical data security access control method based on role-based partially homomorphic encryption user policies. This method indicated that access control policies in cloud environments were more flexible, and attackers could easily collect sensitive data by abusing the access policies of other users. However, the secure access data encryption model developed in cloud environments solves this problem. This model was implemented in Eclipse IDE and AWS Toolkit for Eclipse, and deployed in the Amazon Elastic Beanstalk (EB) environment. It was specifically designed to protect patients’ electronic health details. Although Amazon Elastic Beanstalk provides certain security features, the security of the model also depended on developers’ correct configuration and use of these features. Patil [12] designed a medical IoT security privacy protection and access control method based on password text policy attributes. This method achieved fine-grained control over EHR access, confidentiality, authenticity, and privacy protection by combining the advantages of attribute-based encryption and digital signatures. This method first executed access policies based on the unique characteristics of authorized electronic medical record users, and then supported security functions such as data confidentiality and access authentication through bi-linear pairing. Venema et al. [13] built a data access control method based on attribute encryption, which suggested that data could be stored by entities that were not necessarily trusted to perform access control, or by entities that were not even trusted to access plain-text data. On the contrary, access control could be enforced externally by trusted entities. In addition, some multi-permission variants of ABE (without central permission) can effectively and securely implement access control in multiple domain settings. In addition, ABE is the only encryption method for fine-grained access control, which does not require online trusted third parties during access requests, providing better availability properties. However, the multi-permission variant of ABE is difficult to provide flexible access control without central permission, which increases the complexity of management. Joshi [14] proposed an access control method based on hash algorithm. Hash algorithm was used to hide access strategy and combined signature verification scheme to resist internal attacks, achieving encrypted access to data attributes. However, generally speaking, the hash algorithm is one-way, which means that the original data cannot be recovered from the hash value. Therefore, in some applications, the unidirectionality of hash algorithms may become a problem. For example, hash algorithms cannot provide such functionality when decrypting or restoring encrypted data. In addition, hash collisions (where different inputs produce the same hash value) also pose certain risks. Mittal et al. [15] proposed a secure sharing method for medical data based on blockchain, IPFS, proxy re-encryption, and group communication. Proxy re-encryption and advanced encryption techniques provided strict access control. A blockchain-based group encryption scheme was proposed to ensure the security of group communication. The group session key was agreed upon by authorized group members and used to protect sensitive patient information. Finally, a blockchain data storage based on IPFS was proposed to achieve remote security maintenance of data. Although blockchain technology provides decentralized data storage, its scalability issues can limit the performance of the system. As the group size expands, blockchain may not be able to efficiently handle large amounts of transactions and data storage requirements. Merdassi et al. [16] proposed an LTMA-ABE mobile cloud location and time access security control scheme, which provided fine-grained access control policies based on ABE for encrypted data. When user attributes change, a multi-permission attribute access control system based on user anonymity is proposed to protect user identity from malicious permissions and support permission coexistence. This scheme adopts ABE’s location range constraint as a strategy to authorize users whose dynamic location and time meet these access policies. A multi-permission attribute access control system requires managing complex access control policies, especially when user attributes are dynamically changing. The definition, updating, and maintenance of strategies require a significant investment of computing resources and manpower.

In summary, in terms of data attribute encryption and access control, many scholars and experts have adopted methods such as ABE encryption, hash algorithm encryption, and access control based on blockchain and IPFS, all of which have certain security protection capabilities, but they all have shortcomings. The security of the role based homomorphic encryption user policy method depends on developer configuration and has limited flexibility. Improper configuration can introduce security vulnerabilities; The password text policy attribute method increases system complexity, especially when dealing with a large number of users and permissions, resulting in higher management and maintenance costs for the system; The multi permission variant in attribute encryption methods is difficult to provide flexible access control without central permission; Different inputs in hash algorithms can generate the same hash value, known as hash collision, which can pose certain security risks; The scalability issue of blockchain methods can limit the performance of the system. As the group size expands, blockchain cannot efficiently handle large amounts of transactions and data storage requirements; The dynamic location and time access strategy of LTMA-ABE mobile cloud location and time method can affect the performance of the system, especially in situations where real-time processing of a large number of access requests is required.

The study adopts Empirical Mode Decomposition (EMD) for processing and analyzing high-dimensional data, improving data processability and security. The wavelet thresholding algorithm is introduced for data denoising to improve the quality and readability of encrypted data. Moreover, the study employs fuzzy encryption algorithm to enhance the robustness of the encryption process, ensuring the readability of data even in the event of partial data corruption. Furthermore, a trust level access control attribute tree and an access request graph are constructed, designed to provide fine-grained control over data access. These tools facilitate the management of complex access control policies through a combination of visualization and logical reasoning, thereby simplifying the complexities involved in security management. By seamlessly integrating these technologies, an intelligent encryption access control method tailored specifically for high-dimensional medical data attributes is established, ensuring robust security for such data access.

## 3. Research contribution

Medical data usually contains sensitive personal information such as medical history, diagnosis results, treatment plan, etc. Through attribute encryption technology, fine-grained access control of these data can be achieved, ensuring that only authorized users can access relevant information, so as to protect the privacy of patients. Attribute encryption offers robust support for intricate access control policies. It dynamically adjusts access permissions based on various user attributes, including roles, departments, and permission levels. This ability enables medical institutions to flexibly manage data access based on their specific needs and circumstances. A high-dimensional medical data attribute encryption access control based on fuzzy algorithm is proposed to address the security of existing data encryption access control methods. The main contributions of the proposed method are as follows:

(1) The study incorporates denoising as a mechanism for improving data accuracy, protecting patient privacy, and improving data quality. Through denoising, noise interference during data transmission and storage can be eliminated, ensuring the authenticity and integrity of the data.
(2) Fuzzy encryption algorithm, a novel security technology, is applied to medical data protection. By introducing randomness and fuzziness, the complexity and security of data encryption are significantly improved. In addition, the flexible key configuration strategy proposed in the study can be finely adjusted according to different security levels and user permissions of medical data, ensuring the efficiency and accuracy of data access control. The refined design of the encryption and decryption process further enhances the transparency and security of the data processing, providing an innovative solution for the security management of medical data.
(3) By utilizing dynamic grouping and fuzzy mathematics theory, a comprehensive set of trust calculation methods is designed. These methods aim to optimize medical data access control, ensuring the confidentiality and integrity of the data. Additionally, evaluate the trust relationships between entities and streamline the organizational structure and management mode. Consequently, the methods effectively manage access rights to medical data and significantly reduce the risk of data leakage.
(4) A comprehensive set of advanced technologies is adopted, especially the recursively constructed access request graph, which facilitates dynamic and hierarchical access control. This is complemented by a fine-grained access control strategy aimed at enhancing both security and accuracy. Furthermore, fuzzy encryption algorithm integration enables users to perform keyword fuzzy search while ensuring data security. Additionally, to protect the privacy of the index, an algorithm verification mechanism is implemented to safeguard the privacy and security of the index, thereby preventing keyword information leakage and further strengthening system security and user data privacy protection. The synergistic effect of these innovative technologies ultimately forms an efficient, secure, and user-centric medical data encryption access control framework.

## 4. Proposed method

The detailed technical route of the proposed method is as follows:

Step 1: The first difference of phase data is calculated to evaluate the stability of medical data attributes, and the relative phase change is calculated by combining the relative instantaneous frequency, initial frequency, and linear frequency. EMD algorithm is used to decompose the original medical data into multiple Intrinsic Mode Function (IMF) components and a remainder to remove noise and extract valuable data attributes. The wavelet threshold algorithm is used to reconstruct the high-frequency IMF component twice to achieve more refined denoising effects.
Step 2: On the basis of denoising medical data attributes, this article deeply studies the process of encryption and decryption using fuzzy encryption algorithm. In the encryption stage, the key block size s is set to 24-25. First, data fields are encrypted, followed by record encryption, in which both the system and users can participate to ensure the secure transmission and storage of sensitive information. During the decryption stage, which involves decrypting both records and fields, the process is collaboratively carried out by both the database and the system, ensuring robust data security and identity verification. Furthermore, the modular implementation of encryption algorithms is not constrained by parentheses, thereby enhancing execution efficiency.
Step 3: The trust level access control attribute tree is constructed. Data confidentiality, data integrity, and reputation are considered key characteristics of entity trust to evaluate the trust level of access control objectives. The feedback trust of the recommended nodes is weighted to obtain the recommended trust of the evaluated nodes. The trust relationship, interaction behavior, and data access control objectives between entities are considered to ensure the confidentiality and integrity of access objects.
Step 4: The access request graph is constructed to effectively manage and control users’ encrypted access rights. The graph uses recursive method to combine and layer the associated access request nodes. The subordinate categories that have access to the data are further analyzed, and stringent restrictions are enforced solely on users who possess the corresponding attribute sets and are assigned to the relevant classification category sets, thereby granting them permission to download and decrypt the data. The subordinate categories that have access to the data are further scrutinized, and stringent restrictions are enforced exclusively on users who possess matching attribute sets and classification category sets, allowing them to download and decrypt the data.

The fuzzy encryption algorithm is introduced, which involves the generation of security parameters, user and server keys by the key management center, as well as encrypting files and indexes. After receiving the encrypted data, the server decrypts it with a specific key. When the user searches through keywords, the algorithm will generate a trap door to protect the data privacy.

## 5. Medical data attribute denoising processing

During the transmission and storage of medical data, noise can have adverse effects, leading to phase and frequency deviations in the output data. To enhance the reliability of access control for high-dimensional medical data attributes, it is imperative to undertake denoising processing of medical data attributes. Firstly, denoising can improve data accuracy and integrity, eliminate the impact of noise interference on data attributes, and reduce phases and frequency deviations. This helps to ensure the authenticity of data and improve reliability in data transmission and storage. Secondly, denoising processing helps to protect patient privacy, reduce the risk of sensitive information leakage, and improve the security of encrypted access control systems. Finally, denoising medical data attributes can improve data quality, enhance data interpretability and credibility, ensure that security measures implemented in encrypted access control systems are based on high-quality data, and enhance system reliability and stability. Therefore, medical data attribute denoising is crucial for improving the reliability of access control for high-dimensional medical data attribute encryption, ensuring data accuracy, privacy, and quality, and effectively protecting the integrity and confidentiality of medical data.

Phase data and frequency data are primarily utilized to characterize the stability of the collected medical data attributes. These stability indicators can be derived by calculating the first difference of the phase data and then dividing this difference by the sampling time. The relative change in phase $a\left(t\right)$ and the relative instantaneous frequency deviation $b\left(t\right)$ of medical data attributes are all generated by the determined change in data and random occurrence.

The relative phase change, which is computed under the constraints of random variations, is determined by combining the relative instantaneous frequency, the initial frequency, and the linear frequency component. The specific calculation equation for this relative phase change is presented in equation (1):


$$y\left(t\right)=x\left(t\right)+r\cdot \beta +\frac{k\left(t\right)r}{2}$$ (1)

In equation (1), $x\left(t\right)$ represents the relative instantaneous frequency deviation. *r* represents the frequency data in the initial stage. *β* represents the linear frequency. $k\left(t\right)$ represents the random variation.

The relative phase change $y\left(t\right)$ is obtained according to equation (1), which is constrained by valuable medical data attributes. The original high-dimensional medical data attribute set considering noise data interference is constructed. The specific calculation equation is shown in equation (2):


$$S\left(t\right)=y(t)l\left(t\right)+R\left(t\right)$$ (2)

In equation (2), $l\left(t\right)$ represents valuable medical data attributes. $R\left(t\right)$ stands for noise data.

EMD is a cyclic iterative algorithm, which is conducive to removing noise in medical data through data decomposition. To extract valuable medical data attributes, the EMD algorithm is utilized. This algorithm decomposes the original medical data into multiple IMF components, along with a residual component. Each IMF component represents a specific frequency, and they are arranged in descending order, starting from the largest to the smallest frequency. Importantly, all IMF components must concurrently satisfy the specified conditions:

(1) It must be ensured that the maximum number of points and the number of zeros in each numerical sequence are equal or the maximum difference between the two is 1.
(2) The average envelope of the maximum and the minimum extreme points at a random point is 0.

After EMD decomposition processing, the useful data is relatively stable, while the noisy data is more volatile. Therefore, the high-frequency IMF component of the data is obtained by spectral analysis. The wavelet threshold algorithm is employed to initially reconstruct the acquired high-frequency IMF component twice. Following this reconstruction process, noise reduction is performed, and subsequently, the attributes of the medical data are extracted. The detailed operational steps are outlined below:

Step 1: The number of windows is set as *m*. The swarm intelligence algorithm is used to extract data attributes [17,18]. The amount of data in each window is *J*, and the remaining data is composed into a separate window, with a total of *n* windows.
Step 2: The high-frequency component $e_{1}\left(t\right)$ is separated from the initial medical data attribute S(t). Then, the first residual component $h_{1}\left(t\right)$ is obtained, as shown in equation (3):

$$h_{1}\left(t\right)=S\left(t\right)-z\cdot e_{1}\left(t\right)$$ (3)

In the above equation (3), *z* represents the high-dimensional constraint parameter of medical data attributes.

Step 3: After spectrum analysis and processing of all IMF components are conducted, filtered IMF components that still contain noise are obtained.
Step 4: Soft threshold function and wavelet threshold are used to denoise IMF components containing noise. The corresponding soft threshold coefficient *λ* is shown in equation (4):

$$\lambda = \{\begin{array}{l} \mathrm{sgn}\left(\tau_{k}\right)\left(\left|\tau_{k}\right|-\mu \right),\left|\tau_{k}\right|\geq \mu \\ 0,\left|\tau_{k}\right|<\mu \end{array}$$ (4)

In the above equation (4), *μ* represents the coefficient of determination, and $\tau_{k}$ represents the wavelet transform coefficient.

Step 5: According to the soft threshold coefficient *λ* and the remaining component $h_{i}(t)$ of the *i* -th window, the medical data attribute $Z\left(t\right)$ after denoising processing is obtained, as shown in equation (5):

$$Z\left(t\right)=\sum_{i=1}^{i=m}h_{i}(t)-\lambda R(t)$$ (5)

The above operation calculates the relative phase change by combining relative instantaneous frequency, initial frequency, and linear frequency. The EMD algorithm is used to decompose medical data attributes and filter IMF components containing noise through spectral analysis. Next, the wavelet thresholding algorithm is applied for secondary reconstruction of high-frequency IMF components. Subsequently, a soft thresholding function is employed to perform denoising processing. The soft threshold coefficient is adjusted in order to precisely control the degree of denoising. As a result, the final denoised medical data attributes are obtained, leading to an improvement in the quality of the data attributes and an enhancement of the reliability of access control for high-dimensional medical data.

## 6. Attribute encryption of high-dimensional medical data

Fuzzy Algorithm is a mathematical algorithm based on fuzzy logic, which is used to deal with uncertainty and fuzziness [19–21]. Different from the traditional binary algorithm, fuzzy algorithm can deal with fuzzy input and output, making the calculation result more in line with the actual situation. Traditional encryption algorithms typically rely on deterministic keys for both encrypting and decrypting data. In contrast, fuzzy encryption incorporates elements of randomness and fuzziness, rendering the ciphertext more resistant to cracking. Specifically, a fuzzy algorithm can either generate a fuzzy key or implement a fuzzy encryption approach to bolster data security and enhance resistance against attacks.

### 6.1 Key configuration

**Fig 1:**
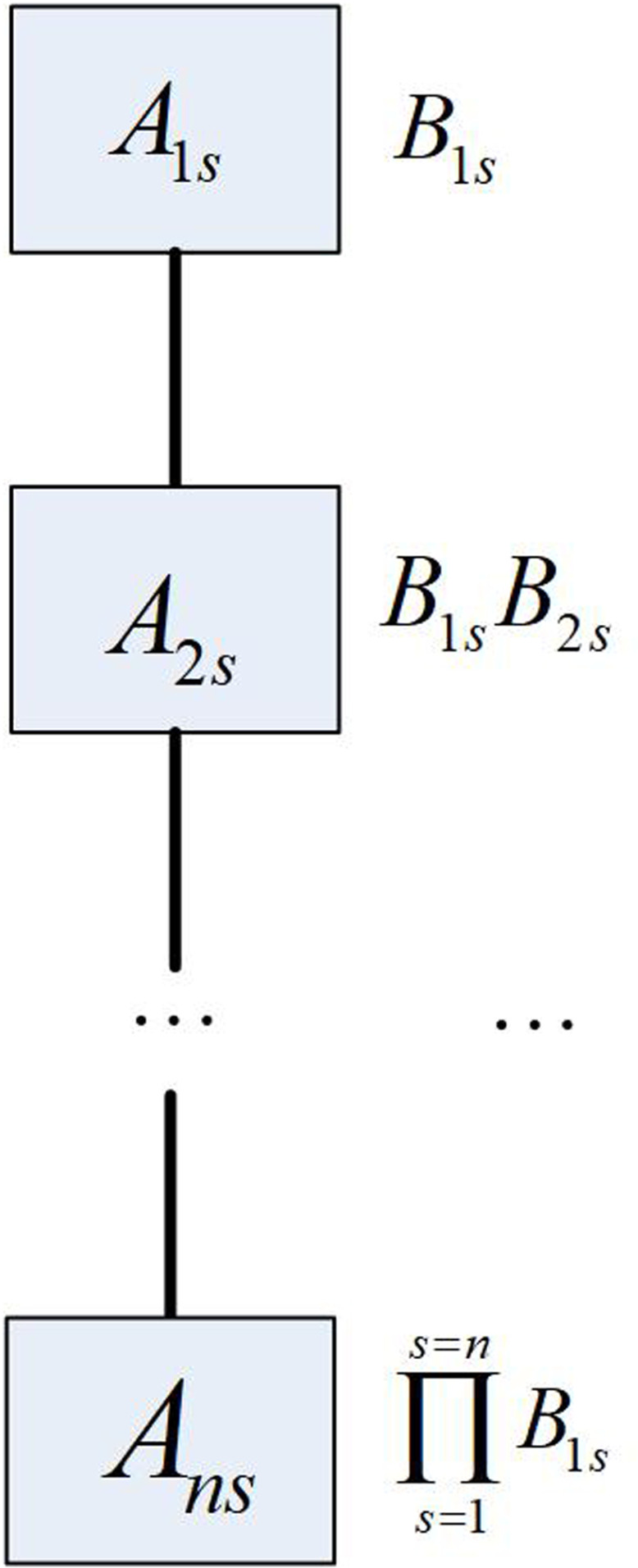
Schematic diagram of medical data security key configuration.

The key configuration of medical data fields with different security levels within the same field is shown in Fig 1.

In Fig 1, the key for the medical data field $A_{ns}$ is $ℵ=\prod_{i=1}^{i=n}B_{ns}$, and 1≤s≤n. $B_{ns}$ represents the sub-key for different fields.

The schematic diagram of medical data security key configuration shows a hierarchical increasing process, introducing new $A_{ns}$ and $B_{ns}$ elements in each stage while retaining all previous stage elements to build a gradually enhanced security mechanism. This configuration ensures high integrity and security of medical data during transmission and storage by continuously accumulating and combining security elements ($A_{ns}$ and $B_{ns}$) at each stage.

For the convenience of describing the encryption process, the intermediate medical data key configuration should be defined to complete encryption calculations or data storage in advance [22,23]. The user security level and field security level are independent of each other. The steps for configuring medical data keys are as follows.

Step 1: User configuration key. The user’s master key is used to decrypt the block key and security level fields, if the user security equivalence is *v*, the key to master decryption is $ℵ'=\prod_{i=1}^{i=n}B_{i}(v)$, where ℵ' represents the security level of the *i* -th data.
Step 2: System configuration key. The system needs to master the block key and the decryption key of all security level fields.

### 6.2 Encryption process

In the encryption process, the threshold for the size of the key block is set to $q_{0}q_{1}$. The size of the medical data key block should be less than $q_{0}q_{1}$ [24,25]. Modular operations are not limited by parentheses. According to the key configuration of medical data security fields, data encryption is implemented. The specific encryption steps are as follows:

Step 1: Field encryption. This step can be implemented not only by users with the same security level as this field, but also by the system. If completed by the user, the ciphertext should be sent to the system after the user performs the encryption operation [26–28]. The specific calculation equation is shown in equation (6):

$$\omega_{i}=Z(t)\frac{c_{i}\cdot q_{0}q_{1}}{K}$$ (6)

In the above equation (6), $c_{i}$ represents the encryption coefficient. *K* represents the security factor.

Step 2: Record encryption. Record encryption is a step that is typically implemented through the use of a system. Specifically, if the security level of each medical data field is equivalent, this step can associate the medical data field with the corresponding security level. The specific calculation method for this association is illustrated in equation (7):

$$\varpi \equiv \sum_{i=1}^{i=n}c_{i}\omega_{i}\mathrm{mod}K$$ (7)

By introducing a threshold for key block size, the encryption process can optimize key partitioning based on actual situations, enhancing performance and security. The rule that modular operations are not limited by parentheses may improve the execution efficiency and adaptability of the algorithm. In addition, the encryption process is customized based on the key configuration of medical data security fields to ensure effective encryption of fields with different security levels.

### 6.3 Decryption process

To assess the security and protection mechanisms of encrypted access control for high-dimensional medical data attributes, it is essential to ensure that the overall data access control system reliably enforces data protection and authentication. Encrypting and decrypting data is a crucial step in preventing unauthorized third-party access to sensitive information that has been collected and stored. The specific decryption steps are as follows.

Step 1: Record decryption. This step is typically handled by the database system itself. However, if the security level of each field within the record is equivalent, users with a corresponding security level may also perform this step. The precise calculation involved in this process is outlined in equation (8):

$$\varpi_{t}=\frac{K}{D}E_{i}\varpi$$ (8)

In the above equation (8), *D* represents the security level reference value, and $E_{i}$ represents the decryption coefficient.

Step 2: Field decryption. This step is usually implemented by the system. If each field has the same security level, this step can also be implemented by users with the same security level as the field.

Firstly, the $\varpi_{t}$ is extracted from *ϖ* to obtain $\varpi_{t}\mathrm{mod}q_{0}$ and $\varpi_{t}\mathrm{mod}q_{1}$. Then, the remainder theorem is used to calculate $\varpi_{t}\mathrm{mod}q_{0}q_{1}$.

The decryption process not only focuses on data recovery, but also emphasizes evaluating and strengthening the security and protection capabilities of the entire encrypted access control system. By implementing a division of labor and cooperation between the database and the system, the decryption process has achieved enhanced efficiency and security. The security level reference value and decryption coefficient together offer flexible and tailored decryption strategies for data that possess varying security levels.

**Fig 2:**
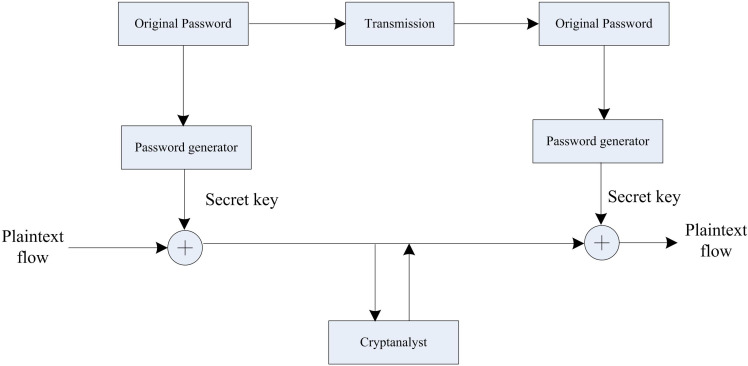
Schematic diagram of encrypted data flow of high-dimensional medical data attributes.

The schematic diagram of the encrypted data flow of high-dimensional medical data attributes is shown in Fig 2.

In Fig 2, the original password is sent from the generating end to the receiving end through a transmission channel, and a key is generated by a password generator at both the generating and receiving ends. Plaintext flow encrypts/decrypts with keys, while Cryptanalyzer is used to analyze and crack encrypted information.

**Fig 3:**
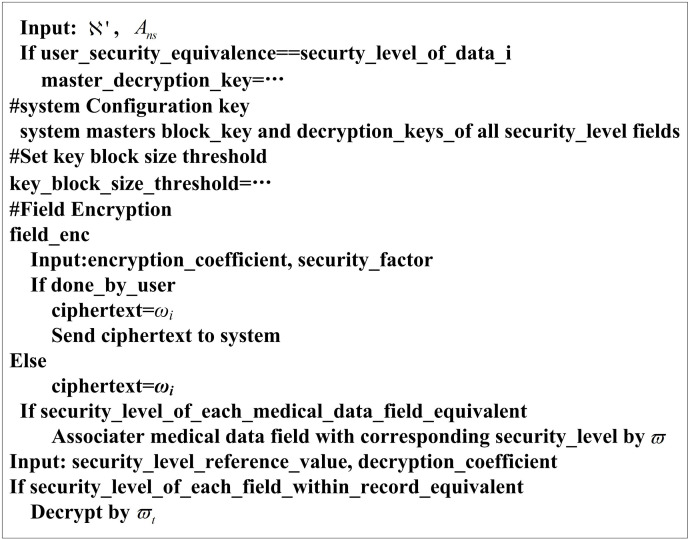
Pseudo-code for encrypting high-dimensional medical data attributes.

The pseudo-code of the attribute encryption algorithm for high-dimensional medical data is shown in Fig 3.

## 7. Medical data attribute encryption access control

After completing the encryption of medical data attributes, in order to further improve the security of the data, it is necessary to further control access permissions.

### 7.1 Trust calculation of access behavior based on dynamic grouping

The logicality between the trust level calculation equations is based on the trust calculation of data access behavior. Firstly, the key characteristics of entity trust are defined, encompassing data confidentiality, data integrity, and reputation. These access control objectives are used as the foundation for trust evaluation. Based on this foundation, the trust access control attribute tree is carefully constructed. Then, the fuzzy mathematics theory is used to establish the trust evaluation level and its mathematical description, and the trust vector calculation equation is designed. The member nodes within the group are classified into different roles based on their common interests. Additionally, an organizational structure tailored for similar interest aggregation, along with a dynamic management mode, is implemented.

**Fig 4:**
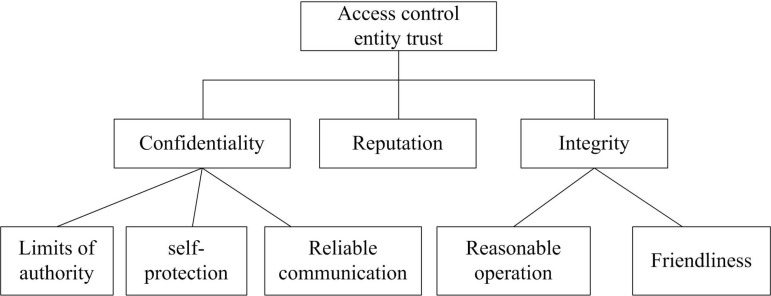
Trust access control attribute tree.

Medical users are considered as unified entities, with access control measures enforced based on their entity trust levels. Additionally, the credibility assessment of these entities’ behavior is specifically aimed at ensuring the confidentiality and integrity of the objects they access. Access control objectives are defined, trust factors of access control for high-dimensional medical data are analyzed layer by layer. The extensible attributes of trust are represented as a tree graph, as shown in Fig 4. The key characteristics of entity trust include data confidentiality, protection data completeness, and credibility [29,30]. Reputation is the judgment of other entities on the trust level of the entity being evaluated, which should be taken as an important element in the trust evaluation process.

The quantitative expression of trust is to build a mathematical model about trust. The model should be constructed according to the unique characteristics of trust, and the reliable mathematical tools should be used to complete the model structure. The inherent subjectivity and fuzziness of trust are pivotal factors influencing the degree of trust and pose a central challenge in accurately describing trust. In this paper, fuzzy mathematics theory is employed to derive an evaluation level and provide a mathematical description of trust, aiming to enhance the overall understanding and representation of trust. $U=\left\{u_{0},u_{1},\cdots ,u_{n}\right\}$ is set to be a collection of connected entities within the medical data. $u_{i}$ is set to be a separate entity. *W* is the trust evaluation set. $C_{W}$ is the evaluation level vector set. The trust vector calculation equation is shown in equation (9):


$$\Phi =\varpi_{t}\mathrm{mod}q_{0}q_{1}\sum_{i=1}^{i=n}\left(C_{W}u_{i}\right)$$ (9)

The grouping concept is integrated into the establishment and assessment of trust models. Consequently, nodes sharing similar interests and hobbies are grouped together. Furthermore, the member nodes within each group are managed in a distributed fashion. By enhancing the frequency of repeated transactions among group nodes, the communication traffic generated by searching for trust data is effectively contained within a localized scope. In turn, the reduction in medical data communication costs leads to enhanced scalability performance of the system. An organizational structure and dynamic management model, centered around the aggregation of similar interests, have been designed. This design categorizes group nodes into three distinct roles: group member nodes, group head nodes, and group manager nodes. The entity node *d* weights the feedback trust of each recommendation node to obtain recommendation data, and obtains the recommendation trust of the evaluated medical data node *f*. According to the calculation results of the trust vector, the recommended trust equation is described in equation (10):


$$\Gamma =\frac{\sum_{I=1}V\left(f\right)}{\Phi \sum_{J=1}T\left(d\right)}$$ (10)

In the above equation (10), $V\left(f\right)$ represents the service trust level of the recommended node towards node *f*. T(d) represents the feedback trust level of node *d*. *I* and *J* represent the recommended node dataset for nodes *f* and *d*.

Under the constraint of recommendation trust, the service trust between member nodes in the same group is calculated, as shown in equation (11):


$$\phi_{fd}=g\cdot +\left(1-\chi \right)g/\Gamma$$ (11)

In equation (11), *χ* represents the frequency parameter, and *g* is the preset threshold for the number of interactions.

The above equation considers the trust relationship between entities, interaction behavior, and data access control objectives, and ensures the confidentiality and integrity of the accessed object. The credibility factor is incorporated into the equation as a crucial component for assessing the trust level of entities. By utilizing weighted calculation methods and setting appropriate frequency parameters, the trust degree is evaluated quantitatively, thereby enhancing the capability to manage and control the trust level during the process of medical data access.

The scenarios that trust calculation can meet include medical data access control, protection of data confidentiality and integrity, establishment and evaluation of trust relationship between entities, optimization of organizational structure and management mode, etc. By calculating and evaluating trust, access rights to medical data can be efficiently managed, thereby mitigating the risk of data leakage.

### 7.2 Access control process

After medical users successfully log in, their access permissions can be analyzed. In order to effectively control the encrypted access permission of the medical user, the access request graph is established. Each concurrent access request in the access graph is regarded as a node. The process of access graph construction is a recursive method, which constructs the associated nodes into an access graph. The process of establishing access requests is as follows:

Step 1: An access graph node is created for parallel medical network access requests.
Step 2: Nodes that triggered access control are categorized into Group (A), while those that do not trigger access control are categorized into Group (B).
Step 3: Group A stratifies nodes according to the control security level of the access resources of the requested node. After stratification, the associated node is created as a new node, which is recorded as an aggregation node.
Step 4: Due to the correlation between levels, the upper and lower nodes are combined together by collecting nodes, and then the newly created collection nodes are connected.

**Fig 5:**
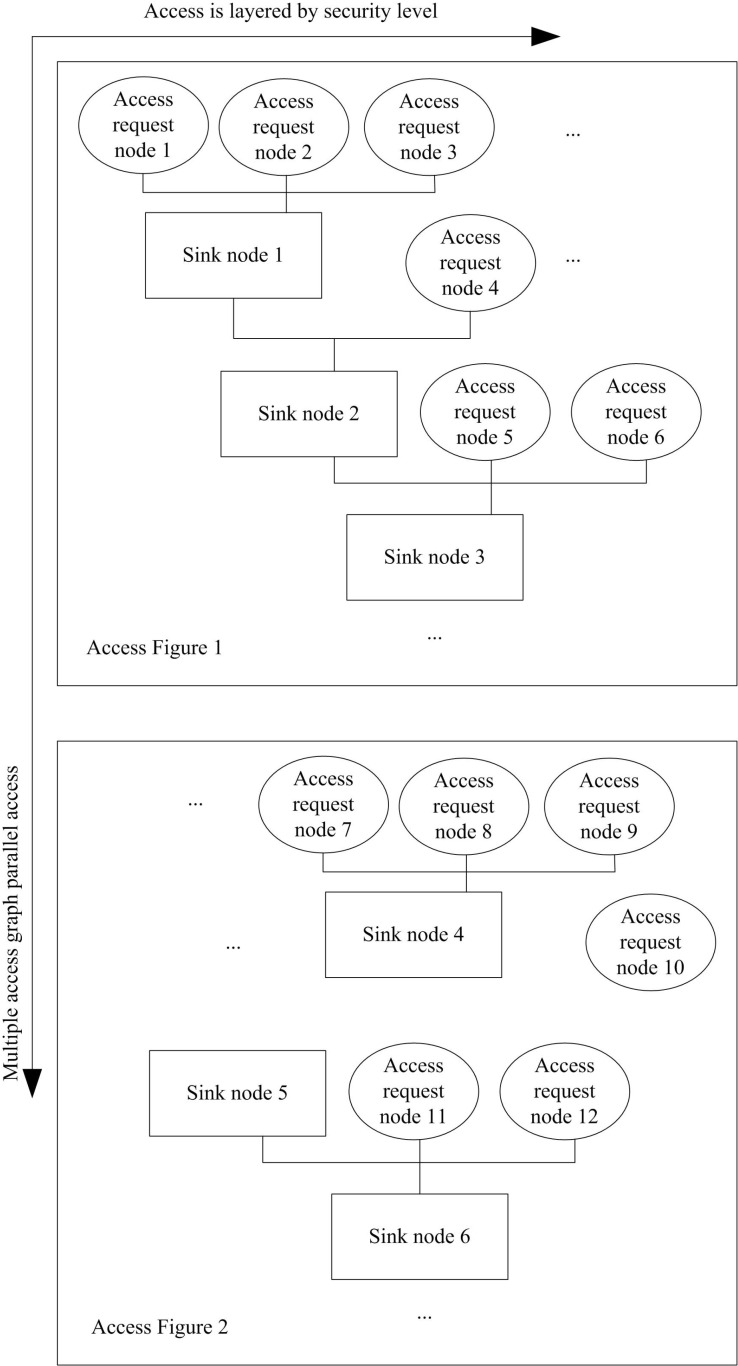
Coordinated medical network access request nodes.

The above steps are repeated to generate a medical network access request, as shown in Fig 5.

Based on the established access request graph, the subordinate categories of the accessed data are analyzed. By examining the dependency classification of accessed data, it can be noted that broad classifications typically include multiple subclasses. Furthermore, due to the interdependence between the accessed data classes, these subclasses can be further divided. Users can only download and decrypt data when they have a universal set of attributes and classification categories that comply with fine-grained medical network access control policies. The classification attributes of the data are determined based on the medical user’s classification category, while the data security level is set according to the user’s security clearance. If the data does not meet the specified requirements, the process will proceed to the next step. Additionally, to further safeguard the security of the data, a fuzzy encryption algorithm is employed to encrypt the data at each layer. Below are the specific encryption processing steps.

Step 1: The key management center takes the security parameter $1^{\gamma }$ as input and outputs a cyclic group *G*, a generator *w* of the cyclic group, a prime number *ρ*, and a hash function *H*. A random trust number *φ* is randomly selected from $\phi_{fd}$ and $\partial =w^{\varphi }$ is calculated. P=(G,w,ρ,∂,H) is output as the public key and Root=φ as the root key.
Step 2: The key management center executes the algorithm to generate user key $Key_{use}$ and server key $Key_{use}$. The user establishes an index $Index=\langle M,File\rangle$ for the high-dimensional medical data attribute keyword *α*, where *M* represents a fuzzy set containing keyword *α* and File represents a file number containing keyword *α*.
Step 3: The fuzzy encryption algorithm needs to encrypt two types of data, namely file File and index Index. The ElGamal proxy encryption algorithm is used to encrypt the file as C(File). Another key $key_{i}$ can be generated based on the user’s private key $key_{use}$ to encrypt the index. The key $key_{i}=H(key_{use})$ is generated through hash function *H*. $Index=\left\{\langle \left[\varsigma ,key_{i}\right]\rangle ,[Enc(key_{i},File)]\right\}$. *ς* is implemented through RSA algorithm, and Enc is implemented through AES algorithm. Finally, the encrypted file and index are uploaded to the server.
Step 4: After receiving the encrypted file and index, the server first finds the server key $Key_{service}$ corresponding to the *i* -th user $Use_{i}$. The final encrypted ciphertext $C*File_{i}$ is obtained.
Step 5: If user $Use_{i}$ searches for relevant encrypted files through keywords, the fuzzy encryption algorithm will generate a trap. The fuzzy set of keywords is $Fuzzy\_M=(M_{1},M_{2},...,M_{n})$. A trapdoor $Fuzzy_{Enc}=\{\varsigma ,(key_{i})\}$ is generated through the key $key_{i}$, and the user sends $Fuzzy_{Enc}$ to the server.
Step 6: After receiving the trapdoor $Fuzzy_{Enc}$ sent by the user, the server will compare $Fuzzy_{Enc}$ with the index and return all encrypted files $Enc(key_{i},File)$ that meet the conditions. The server decrypts $Enc(key_{i},File)$ to obtain $File_{i}$, which is also the relevant encrypted file $C*(File_{i})$.
Step 7: The server first finds the server key $Key_{service1}$ corresponding to user $Use_{i}$ stored on it, and uses $Key_{service1}$ to partially decrypt $C*(File_{i})$. Therefore, the ciphertext becomes $C'(File_{i})$, and the server sends $C'(File_{i})$ to user $Use_{j}$.
Step 8: After receiving the cipher-text $C'(File_{i})$ sent by the server, user $Use_{j}$ uses the key in their hands to calculate $File_{i}$ and get the final civilization file.

In the fuzzy encryption scheme presented in this article, the index calculation remains consistent for requests pertaining to the same keyword, thus necessitating the proof of only the privacy and security of the index. Assuming that the search scheme cannot guarantee the privacy and security of the index in the case of keyword attacks, there exists an algorithm *ϑ* that can extract some potential information of keywords from the index. This article establishes ϑ' to use algorithm *ϑ* to determine whether certain functions δ'(.) are pseudo-random functions. The algorithm ϑ' has $OracleO_{\delta '(\cdot )}$ permission and can take a secret value *κ* as input. Then, δ'(κ) is returned. After receiving a request for index calculation, ϑ' sends the δ'(κ) request to $OracleO_{\delta '(\cdot )}$. After completing the trapdoor query, the attacker will output two keywords $\iota_{0}$ and $\iota_{1}$ with the same length and distance. ϑ' randomly selects a σ∈{0,1}, and sends $\iota_{\sigma }$ to the challenger. Then, ϑ' obtains a value *Y* generated by a pseudo-random function δ(.) or a random function. ϑ' sends the value *Y* to *ϑ*, and *ϑ* responds to ϑ' with σ'∈{0,1}. If *ϑ* can correctly guess the value of $\iota_{\sigma }$ with an undeniable probability, it indicates that the value *Y* is not randomly generated. Therefore, ϑ' can determine whether δ'(.) is a pseudo-random function. Due to the indistinguishability between pseudo-random functions and real random functions, the maximum probability of *ϑ* correctly guessing the value of $\iota_{\sigma }$ is 1/2. Therefore, the privacy search is secure.

Based on the aforementioned steps, the medical network access can swiftly pinpoint black hole information as well as detect potential malicious intrusion risk details. The medical user access situation is planned based on the above process. Concurrent users are managed efficiently, and the risk of medical data leakage due to simultaneous malicious access is mitigated. This holistic approach guarantees the successful implementation of encryption access control for high-dimensional medical data attributes.

**Fig 6:**
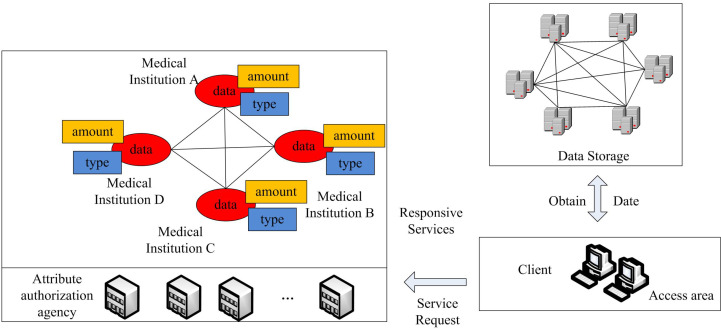
Access control block diagram based on fuzzy algorithm.

The access control block diagram based on fuzzy algorithm is shown in Fig 6.

In Fig 6, multiple medical entities interact with each other in a complex and orderly manner. The datasets handled by these medical entities undergo meticulous classification into “amount,” “type,” and “specific data content.” Each category is entrusted to a rigorously authorized central management agency to maintain compliance and security in data transmission and processing. In the data storage and access system, the data storage system acts as a pivotal element. It retrieves and prepares data resources from highly secure storage devices. This process facilitates the system’s ability to efficiently meet a wide range of client demands. Within the data storage and access system, clients submit service requests via specifically designed access points. After receiving these requests, the system quickly and accurately provides tailored data service responses for the requested content. This arrangement establishes a seamless closed-loop data request-response process. It not only facilitates efficient data flow, but also strengthens security measures around data access.

**Fig 7:**
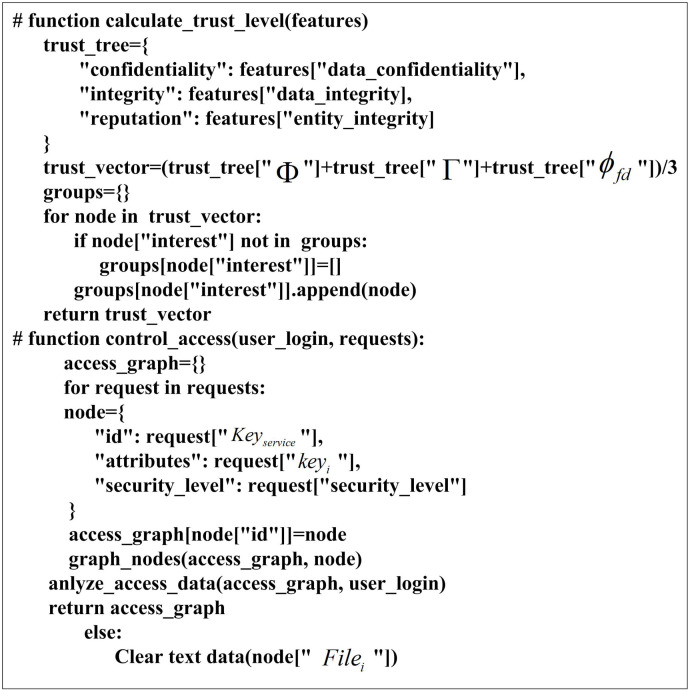
Access control pseudo-code.

The pseudo-code for access control is shown in Fig 7.

## 8. Experimental verification

In this experiment, the data attribute encryption access control process is divided into the following steps.

Step 1: The access control method is run to obtain private and public keys;
Step 2: The method is connected to data attributes, statistical data is summarized, and the attributes and attribute weights of the data stored in the data attributes are calculated;
Step 3: The attribute weights are determined, an access control model is generated, and the data that needs to be encrypted is encrypted to obtain cipher-text;
Step 4: The data attribute set is determined and a marked user private key is generated;
Step 5: The cipher-text is decrypted to obtain access to data attributes.

The computer configuration used in the experiment is Intel Core i7-5775C, CPU. It has 8 cores at 2.1GHz, 16GB of memory, and a 2T hard drive. In terms of testing indicators, access time and encryption/decryption duration are selected to test the computational efficiency of the proposed method. The stability of the proposed method is verified using packet loss rate, and the security performance is tested using attack success rate. The settings of each parameter in the experiment are shown in Table 1

**Table 1:** Experimental parameter settings.

Parameter	EDM	Wavelet thresholding algorithm
Decomposition level	15	/
Relative tolerance	1.1	/
Maximum number of iterations	100	/
Maximum number of IMF	16	/
Maximum number of extreme values	1	
Specific energy	8	
Decomposition level	/	7
Wavelet basis	/	Symlets

### 8.1 Access time test results

**Fig 8:**
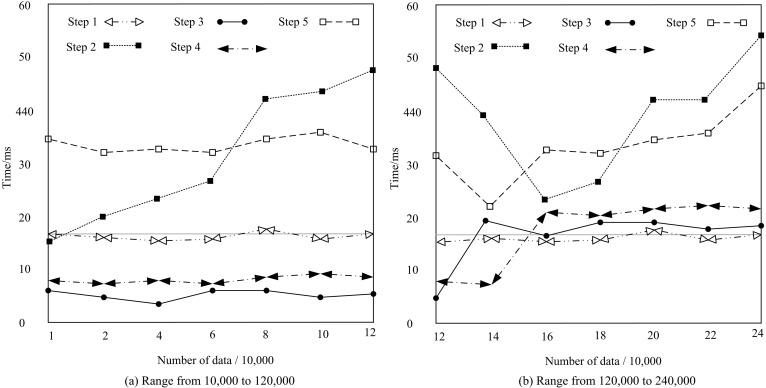
Access time test results.

In real-world applications, high-dimensional medical data often encompasses a vast number of attributes and records, resulting in a significant volume and complexity of data. If the performance of the encrypted access control method is slow or inefficient, users may encounter delays or experience prolonged response times when accessing and manipulating this data. This has a direct impact on user experience and productivity. The medical field has high requirements for real-time and timely response of data. For example, in a clinical decision support system, doctors need to quickly access and analyze data to make accurate diagnosis and treatment recommendations. If the time consuming performance of encrypted access control methods is too high, it may not be able to meet the needs of real-time data processing and response. The number of medical data is set to gradually increase from 10,000 pieces to 1 million pieces. The calculation time of the proposed method is recorded, as shown in Fig 8.

As can be seen from Fig 8, when the proposed method stored a small amount of data in the data attributes, the operation time required for each stage remained basically unchanged. However, when the amount of data stored in the data attributes increased, the data for statistical induction of data attributes and their weight values increased sharply, resulting in a significant increase in the computation time of step 2. When the number of data attributes reached 240,000, the encrypted access control time was still less than 60ms. The results show that the proposed method has a fast operation efficiency when controlling data attribute access.

### 8.2 Data attribute encryption - time comparison test of different decryption methods

**Fig 9:**
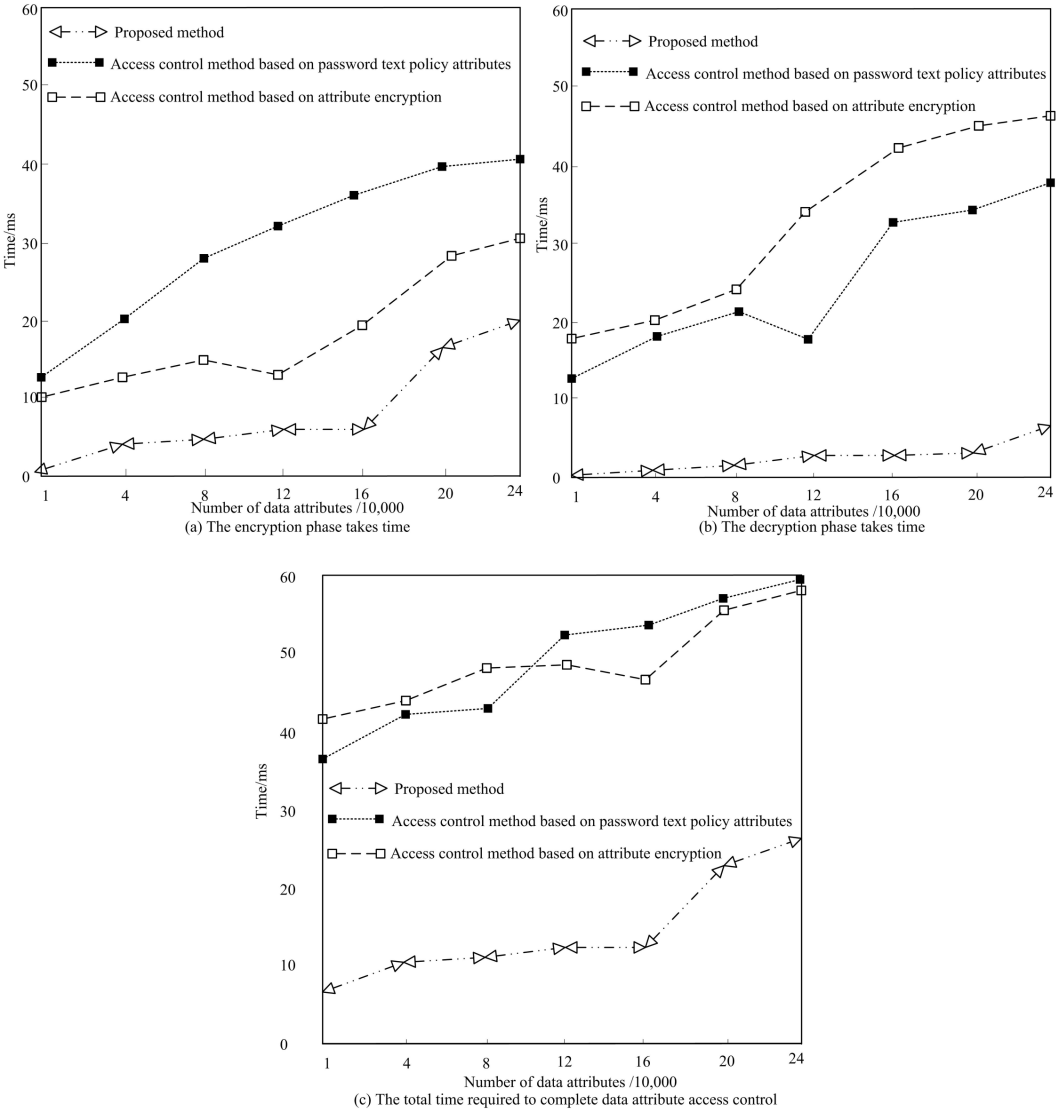
Comparison of encryption and decryption time using different methods.

The access control method based on password text policy attributes [12] and the access control method based on attribute encryption [13] are used as comparison methods. The encryption, decryption, and total control time of its data are compared with the proposed method. The results are shown in Fig 9.

From Fig 9, as the number of data attributes increased, the encryption and decryption time of the three sets of access control methods also increased. The encryption and decryption time of the proposed method notably surpasses those of access control methods based on password text policies and attribute encryption. Consequently, this method is unlikely to bring additional operational pressure to the system when managing data attribute access.

### 8.3 Comparison of packet loss rate under different methods

During the transmission of high-dimensional medical data, packets may be lost or corrupted due to network or communication equipment failures. If the encrypted data is lost or damaged, it cannot be decrypted correctly and the data cannot be recovered. The high-dimensional medical data often has a large volume and complex structure, and it needs a large amount of computing resources to encrypt it with strong encryption algorithm. During the process of data transmission, the encryption operation may experience excessive delays due to limitations in communication bandwidth or processing capabilities. Consequently, the data may not be transmitted in a timely and efficient manner, ultimately resulting in potential data loss.

**Fig 10:**
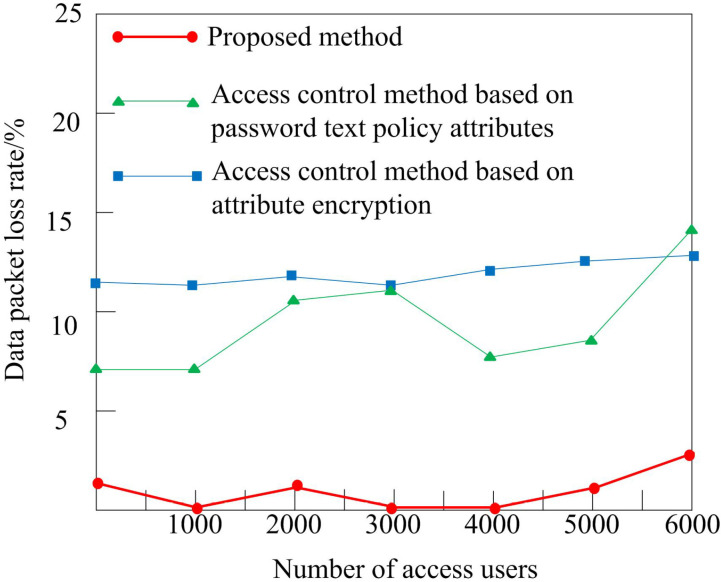
Comparison of data packet loss rates.

For this reason, in this experiment, the data packet loss rate is taken as an indicator of the comparison method. The packet loss rate refers to the proportion of packets lost during data transmission, usually expressed as a percentage. The comparison results are shown in Fig 10.

As shown in Fig 10, the high-dimensional medical data attribute encryption access control method in this study showed a significant reduction in packet loss rate after control, almost eliminating the situation of packet loss. In contrast, access control methods that rely on password text policy attributes and attribute encryption exhibit a higher frequency of packet loss during the control process, indicating that the control method explored in this study shows excellent performance in terms of application effectiveness.

### 8.4 Different methods of attack success rate test

The main attack faced in high-dimensional medical data attribute encryption access control is “privacy breach attack”. This is because high-dimensional medical data may contain sensitive privacy information of patients, such as case records, diagnostic results, treatment plans, etc. If data that lacks effective encryption and access control measures is leaked, the privacy of patients will be compromised. Attackers may endeavor to deduce the identity and sensitive information of individual patients by exploiting correlations between multiple anonymized datasets. There are a large number of attributes in high-dimensional data, which can make it easier to identify and correlate. Therefore, in this test, the type of attack designed is privacy breach attack.

By testing the success rate of resisting attacks, the security of the system can be verified and effectively defended against attacks, protecting data from leakage or damage. The success rate of resisting attacks is shown in equation (12):


$$l=\frac{\psi_{ϒ}}{\psi_{total}}\times 100\%$$ (12)

In equation (12), $\psi_{ϒ}$ represents the number of successful attempts to resist attacks, and $\psi_{total}$ represents the total number of attacks.

**Fig 11:**
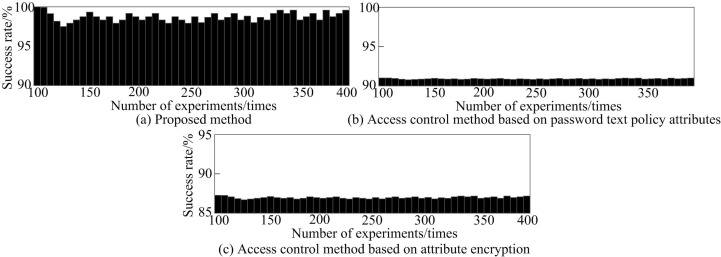
Comparison of success rates of different methods in resisting attacks.

During the process of encrypting access to medical data attributes, a human privacy breach attack is carried out on all medical data. The success rates of resisting attacks using different methods are tested. The experimental results are shown in Fig 11.

From Fig 11, the success rate of using the encryption method to resist attacks was significantly higher than the other two methods, with a maximum of 100%. The highest success rates of access control methods based on password text policy attributes and attribute encryption to resist attacks were 91.5% and 87.5%, respectively. It can be seen that the proposed method has better security and can effectively prevent the leakage of medical data attributes.

## 9. Discussion

Based on the above research results, firstly, although the computation time increases when the data volume is large, the entire encryption access control time is still less than 60ms when the number of data attributes reaches 240000. This result indicates that even in the face of relatively large medical datasets, the proposed method can still maintain fast computational efficiency, meeting the high requirements of the medical field for real-time data processing and timely response. Secondly, as the number of data attributes increases, the encryption and decryption time of the three access control methods all increase, but the proposed method has significantly lower encryption and decryption and total control time than comparison methods. In the encryption stage, the longest time consumption of the proposed method is only 20ms, which is 21ms less than the access control method based on password text policy attributes, indicating that the proposed method has a lower operational burden in controlling data attribute access. After that, in high-dimensional medical data transmission, the data attribute encryption access control method proposed in this study significantly reduces the data packet loss rate, with almost no packet loss phenomenon. In the following section, the medical data attribute encryption access control method introduced in this article demonstrates a significantly higher success rate in preventing human privacy leakage attacks, when compared to traditional methods that rely on password text policy attributes and attribute encryption. The proposed method has a maximum success rate of 100% in resisting attacks, which is 8.5% and 12.5% higher than other methods, effectively enhancing the security of medical data. Finally, the experiment shows that the method is still effective when the number of data attributes reaches millions.

## 10. Conclusion

It is very important to ensure that only authorized users can access high-dimensional medical data. Therefore, it is necessary to design appropriate user authentication and access authorization mechanisms to ensure that legitimate users can access and manipulate data. Therefore, this study proposed a high-dimensional medical data attribute encryption access control method based on fuzzy algorithm. The EMD algorithm effectively removed noise from medical data, improving the quality and reliability of the data. In order to strengthen data protection, a fuzzy encryption algorithm was designed for key configuration, effectively preventing unauthorized access and data leakage. Finally, by calculating the credibility of access behavior based on dynamic grouping, data access permissions could be more accurately controlled. The test results showed that the proposed method effectively resisted privacy leakage attacks. The proposed method had a success rate of up to 100% in resisting attacks. Although the high-dimensional medical data attribute encryption access control method based on fuzzy algorithm proposed in this study shows good performance in experiments, there are still some limitations, such as the complexity of user authentication and access authorization mechanisms, which can increase the difficulty of implementation. In future research endeavors, the focus will be on simplifying user authentication and access authorization mechanisms, while ensuring strong data security to enhance their deployability and maintainability.
